# Health Risk Assessment during In Situ Remediation of Cr(VI)-Contaminated Groundwater by Permeable Reactive Barriers: A Field-Scale Study

**DOI:** 10.3390/ijerph192013079

**Published:** 2022-10-12

**Authors:** Wenjing Zhang, Yifan Zhu, Ruiting Gu, Zhentian Liang, Wenyan Xu, Muhammad Yousuf Jat Baloch

**Affiliations:** 1 Key Laboratory of Groundwater Resources and Environment, Jilin University, Ministry of Education, Changchun 130021, China; 2 College of New Energy and Environment, Jilin University, Changchun 130021, China; 3 School of Earth Sciences and Engineering, Nanjing University, Nanjing 210023, China; 4 Chemical Geological Prospecting Institute of Liaoning Province Co., Ltd., Jinzhou 121007, China

**Keywords:** groundwater, health risk assessment, Cr(VI) contamination, permeable reactive barriers, field-scale

## Abstract

The presence of residual Cr(VI) in soils causes groundwater contamination in aquifers, affecting the health of exposed populations. Initially, permeable reactive barriers(PRB) effectively removed Cr(VI) from groundwater. However, as PRB clogging increased and Cr(VI) was released from upstream soils, the contamination plume continued to spread downstream. By 2020, the level of contamination in the downstream was nearly identical to that in the upstream. The study results show that during normal operation, the PRB can successfully remove Cr(VI) from contaminated groundwater and reduce the carcinogenic and non-carcinogenic risks to humans from the downstream side of groundwater. However, the remediated groundwater still poses an unacceptable risk to human health. The sensitivity analysis revealed that the concentration of the pollutant was the most sensitive parameter and interacted significantly with other factors. Ultimately, it was determined that the residual Cr(VI) in the soil of the study region continues to contaminate the groundwater and constitutes a serious health danger to residents in the vicinity. As remediated groundwater still poses a severe threat to human health, PRB may not be as effective as people believe.

## 1. Introduction

Groundwater is one of the most commonly used water supplies, but microbiological and chemical pollution contaminate groundwater [1,2]. Chromium (Cr) in groundwater has been a severe worldwide health and environmental problem for many years [3]. Cr pollution in the environment is mainly the result of human activities such as electroplating, leather tanning, textile production, and metallurgy [4,5]. These industries produce significant quantities of wastewater, including solid sludge and chromium-containing waste [6]. Cr is a primary heavy metal used in the electroplating industry, mainly existing as Cr(VI), and it is hazardous to human health and the environment [7]. There is also a portion of chromium pollution originating in nature, with some minerals containing significant amounts of Cr(III), which are oxidized to Cr(VI) during weathering and are highly mobile [8]. Cr(VI) levels in groundwater at specific contaminated locations in China have reached as high as 600 mg/L [9]. The primary valence states of chromium in the environment are trivalent (Cr(III)) and hexavalent (Cr(VI)) ionic groups [10]. Cr(VI) is more toxic than Cr(III) and easily enters human bodies via different routes, including inhalation ingestion, or even directly from the skin and then transferred to organs such as lungs, kidney, liver, carcinogenic, mutagenicity, genotoxicity and teratogenic. At the same time, Cr(III) is more stable, simpler to precipitate, easier to remove from groundwater, and less hazardous than Cr(VI) [11]. Numerous studies have demonstrated that Cr absorbed by crops from the soil can translocate and concentrate in the areal plant parts. Cr (VI) can produce oxidative imbalances and mutagenesis, reduce biomass, inhibit flowering, fruit set, crop yield loss, and degrade grain quality [12,13]. There is a significant association between exposure to high levels of Cr(VI) in contaminated wastewater, drinking groundwater, soil, and crops and a 60-fold increase in the risk of stomach cancer [14]. Long-term exposure to Cr(VI) can also cause skin lesions, ulcers, nasal septum perforations, perforated eardrums, decreased sperm production, and lung cancer [15,16,17]. Therefore, the risk posed by Cr(VI) contaminated locations to humans should be highly considered.

Depending on the severity of the Cr(VI) danger, various remediation strategies have been devised for various polluted sites. Physical, chemical, biological, and a mix of these techniques are common remediation methods for Cr(VI)-contaminated groundwater [18,19]. Permeable reactive barriers (PRB) are more cost-effective than other in situ remediation approaches for groundwater contamination cleanup [20]. PRB is a passive treatment technique that uses reactive media to remove contaminants from groundwater, and it may successfully immobilize or degrade several metals, metalloids, and organic pollutants [21]. The active media is the filling material within the PRB, and it is one of the reactants for function-based contaminant treatment. Removing Cr(VI) with reactive materials such as zero-valent iron (ZVI), activated carbon, natural minerals, and nanomaterials has been demonstrated in studies [22]. Because of its inexpensive cost, great reduction potential, and high reactivity, ZVI is the most extensively utilized reactive material in PRB [23,24]. Under certain conditions, laboratory and field experiments have shown that reaction media delivered underground by PRB successfully convert or immobilize Cr(VI) for a relatively long time [25,26]. However, PRB cannot remove elemental Cr from groundwater, and Cr(III) is slowly oxidized to Cr(VI) in groundwater [27]. Consequently, the risk of human health in the process of groundwater remediation by PRB is uncertain, and scientific control of the contamination risk of Cr-contaminated sites during the entire remediation cycle is a crucial factor in determining the applicability of the techniques.

The United States Environmental Protection Agency (US-EPA) publishes the human exposure assessment guidelines widely utilized internationally to evaluate health risks [28,29,30]. The Ministry of Ecology and Environment People’s Republic of China (MEEPRC) of China has re-evaluated the relevant parameters of the human exposure assessment guidelines in light of the current situation in China and developed similar guidelines for health risk assessment; Chinese academics have published studies employing these guidelines [31]. Cr is a common heavy element in groundwater and drinking water contaminated with Cr(VI) can significantly impact the health of people in the area [32,33,34]. Therefore, it is important to conduct a human health risk assessment of groundwater for the health of the residents in the study area. When human health hazards are involved in a health risk assessment, it is crucial to examine their essential parameters. Due to the uncertainty in the input model parameters, the consideration of parameter values is especially significant [35]. To overcome these problems, this work employed Sobol sensitivity analysis to estimate the sensitivity of the risk assessment model’s parameters. Sobol sensitivity analysis is a variance decomposition technique used to comprehend the influence of model outputs that account for input sensitivity [36]. This method has also been applied to determining sensitivity parameters for human health risks in previous studies [37,38,39].

Given the varying levels of Cr(VI) pollution in groundwater caused by different PRB operating circumstances at different times in the research area, and risk assessment guidelines applicable to China for assessing the health risk posed by groundwater. This study aimed to (1) determine the concentration and spatial distribution of Cr(VI) in groundwater resources at a contaminated site located in the North China Plain using a Geographic Information System (GIS) and analyze the effect of PRB on Cr(VI) in groundwater; (2) conduct a health risk assessment of Cr(VI) contaminated soil and groundwater in the study area using the Technical Guidelines for Risk Assessment of Soil Contamination of Land for Construction developed by MEEPRC (Ministry of Ecology and Environment of the People’s Republic of China); and (3) analyze a model of adult carcinogenic risk in the study area using Sobol sensitivity analysis, considering three indicators of first-order effects (FOE), second-order effects (SOE), and total effects (TE). The study results provide essential theoretical and practical references for improving remediation technology, managing industrial waste, and protecting human health at sites contaminated by Cr(VI) in China.

## 2. Materials and Methods

### 2.1. Study Area

**Figure 1:**
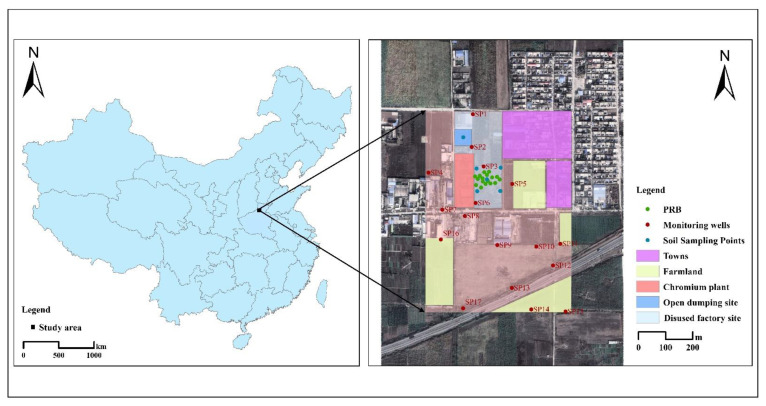
Location map of the study area and groundwater sampling locations.

The research area is situated in the North China Plain and encompasses over 400,000 m^2^, as shown in Figure 1. The area under study has a continental monsoon climate with predominant northeasterly winds in the winter and southwesterly winds in the summer. The average annual precipitation is 656mm, from June to September, accounting for 72% of the average annual precipitation. The average annual evaporation is 1748 mm, while the average annual temperature is 14 °C. The geography in the research region consists primarily of an alluvial plain with a flat topology and a gentle slope from northwest to southeast, with a ground elevation between 50.73 and 52.84 m and a hydraulic slope of 2.2–3.2‰. Groundwater flow direction in the study area is from North North-East (NNE) to South South-West (SSW).

In the studied area, an abandoned facility mainly produces chromium salt. The chrome salt production factory was located in the southern portion of the property, and the chrome waste was heaped approximately 100 m to the north of the plant. In the 1980s, production ceased, and the remaining chrome slag covered an area approximately 4900 m^2^. In the 1990s, the chrome salt plant was converted into a phosphate-producing chemical plant, and the chrome salt production facility was replaced with guest soil during construction. During excavation, a huge amount of yellow stuff was discovered in the soil. The local environmental protection agency (EPA) disposed of the remaining chrome slag in 2009, and by 2014, the chrome garbage stacked above the ground had been eliminated, but there were still remnants. Groundwater pollution PRB remediation project was initiated at the contaminated site in 2017, and PRB expired in 2019. The facility is surrounded by the agricultural land of a town where primarily wheat and maize are farmed.

### 2.2. Sample Collection and Analysis

The groundwater samples were collected in September 2016, April 2018, January 2019, and May 2020, using 17 groundwater monitoring wells and PRB within the abandoned plant. In May 2020, six soil samples were gathered from within the abandoned facility. The sampling process conducted in triplicates at each site to ensure. Clean 1-liter high-density polyethylene bottles were utilized for groundwater sampling; the bottles were rinsed three times with double-distilled water prior to sampling and dried by the oven. The pH of the obtained water samples was lowered to below 2 with ultra-pure nitric acid (ultra-pure > 99%), and the water samples were then sent to the laboratory. The samples were analyzed 1,5-diphenylcarbazide(DPC) spectrophotometer method to determine the Cr concentration three times in the laboratory [40]. The spectrophotometer (Unic 7200) were used for the experiments. Polymethyl methacrylate (PMMA) cuvettes with an optical range of 1 cm were used throughout the experiments. All the chemicals were used of analytical grade. The DPC method for Cr(VI) determination was conducted with the guidelines of the GB 7467-87 standard (China). The top 10 cm of soil at the sampling spot were removed prior to sampling, and an additional 30 cm of soil were collected for analysis. These samples were packed in clearly labeled 2.5 L clear polythene bags and transferred to the laboratory in a 10 °C freezer for analysis. All obtained soil samples were air-dried, and subjected to acid dissolution, and the Cr concentration was measured using the DPC method. The UV spectrophotometer was calibrated beforehand to ensure accurate analytical results from the experimental apparatus, and sample solutions were prepared using standard procedures.

### 2.3. Human Health Risk Assessment

The health risk assessment adheres to the 2019 MEEPRC. Technical Guidelines for Risk Assessment of Soil Contamination of Land for Construction are comparable to the US-EPA Metals Risk Assessment Guidelines [31]. Based on the duration of adult exposure, industrial locations with sensitive receptors were chosen as exposure scenarios to evaluate the carcinogenic and non-carcinogenic risk of pollutants. Since chromium does not exist as a gaseous contaminant, there is no exposure pathway for inhalation of airborne gaseous contaminants volatilized from soil or groundwater; therefore, ingestion of soil, dermal contact with soil, inhalation of soil particulate matter, and consumption of groundwater were considered as potential exposure routes.

#### 2.3.1. Exposure Routes and Calculating Exposure

The exposure doses under the four different exposure routes are calculated using the following Equations (1)–(4).

(1) $${\mathrm{ER}}_{\mathrm{ing}}=\frac{{\mathrm{OSIR}}_{a}\times {\mathrm{ED}}_{a}\times {\mathrm{EF}}_{a}\times {\mathrm{ABS}}_{\mathrm{ing}}}{{\mathrm{BW}}_{a}\times \mathrm{AT}}\times 10^{-6}$$

(2) $${\mathrm{ER}}_{\mathrm{derm}}=\frac{{\mathrm{SAE}}_{a}\times {\mathrm{SSAR}}_{a}\times {\mathrm{ED}}_{a}\times {\mathrm{EF}}_{a}\times E_{v}\times {\mathrm{ABS}}_{\mathrm{derm}}}{{\mathrm{BW}}_{a}\times \mathrm{AT}}\times 10^{-6}$$

(3) $${\mathrm{ER}}_{\mathrm{inh}}=\frac{{\mathrm{PM}}_{10}\times {\mathrm{DAIR}}_{a}\times {\mathrm{ED}}_{a}\times \mathrm{PIAF}\times \left(\mathrm{fspo}\times {\mathrm{EFO}}_{a}+\mathrm{fspi}\times {\mathrm{EFI}}_{a}\right)}{{\mathrm{BW}}_{a}\times \mathrm{AT}}\times 10^{-6}$$

(4) $${\mathrm{ER}}_{\mathrm{dg}}=\frac{{\mathrm{GWCR}}_{a}\times {\mathrm{EF}}_{a}\times {\mathrm{ED}}_{a}}{{\mathrm{BW}}_{a}\times \mathrm{AT}}$$

where ER is the exposure under different exposure routes (mg × kg^−1^ × d^−1^, ing, derm, inh and dg represents four exposure routes); ${\mathrm{OSIR}}_{a}$ is the daily oral intake of soil for adults (mg/day); ${\mathrm{ED}}_{a}$ is the exposure duration of adults (a); ${\mathrm{EF}}_{a}$ is the exposure frequency of adults (d/a); ABS is the absorption factor of different exposure routes; ${\mathrm{BW}}_{a}$ is the weight of adult (kg); AT is the average time for carcinogenic or non-carcinogenic effects (d); ${\mathrm{SAE}}_{a}$ is the surface area of exposed skin for adults (cm^2^); ${\mathrm{SSAR}}_{a}$ is the soil adhesion factor for adult skin surfaces (mg × cm^−2^); $E_{v}$ is the daily frequency of dermal contact events (freq × d^−1^); ${\mathrm{PM}}_{10}$ is the level of respirable particulate matter in the air (mg × m^−3^); ${\mathrm{DAIR}}_{a}$ is the daily volume of air breathed by an adult (m^3^ × d^−1^); PIAF is the proportion of inhaled soil particulate matter retained in the body; fspo is the proportion of particulate matter in outdoor air that comes from soil; ${\mathrm{EFO}}_{a}$ is the frequency of outdoor exposure for adults (d × a^−1^); fspi is the proportion of particulate matter in indoor air that comes from soil; ${\mathrm{EFI}}_{a}$ is the frequency of indoor exposure for adults (d × a^−1^); ${\mathrm{GWCR}}_{a}$ is the daily adult water intake (L × a^−1^).

#### 2.3.2. Risk Characterization and Contribution Rates

The carcinogenic risk (CR) and non-carcinogenic hazard quotient (HQ) are calculated using the following Equations (5) and (6)

Cancer risk and hazard quotient calculating formulas for four soil exposure routes.

(5) CR=ER×C×SF

(6) $$\mathrm{HQ}=\frac{\mathrm{ER}\times C}{\mathrm{RfD}\times \mathrm{AF}}$$

where CR is the risk of cancer from different exposure routes; HQ is the non-carcinogenic hazard quotient for different exposure routes; C is the concentration of a contaminant in soil or groundwater (mg/kg or mg/L); SF is a slope factor for carcinogenicity for different modes of exposure to pollutants, including ingestion, dermal exposure and inhalation; AF is the reference dose distribution factor for exposure to soil or groundwater; RfD is a non-carcinogenic reference dose for different modes of exposure to pollutants, including ingestion, dermal exposure and inhalation (mg/(kg day)).

The risk contribution of the different exposure routes is calculated using Equation (7). There is only one exposure route for contaminants in groundwater for drinking; therefore, no contribution calculation has been made.

(7) $$R_{i}=\frac{{\mathrm{CR}}_{i}}{\sum {\mathrm{CR}}_{i}}$$

where ${\mathrm{CR}}_{i}$ is the calculated carcinogenic risk for different exposure pathways of contaminants in soil, $R_{i}$ is the risk contribution of the i-th exposure route. This formula can also calculate the non-carcinogenic risk contribution of the different exposure routes.

### 2.4. Sensitivity Analysis

Sobol sensitivity analysis is a sophisticated technique for determining whether responses and their processes have additional effects on the overall system by evaluating the relative extent of each significant input and its interaction with the output model’s variance [35]. Sobol sensitivity analysis is extensively employed in health risk assessment, and complete evaluation methodologies have been described in the pertinent papers [35,38,39]. When the sensitivity index of the input parameters is >0.1, 0.01–0.1, and 0.01, they are categorized as very sensitive, sensitive, and insensitive parameters, respectively [38]. In this work, we performed 10,000 iterations of sensitivity analysis on the parameters of the health risk assessment of Cr(VI) pollution in soil and groundwater in the study area using the SALib module in Python 3.8.

### 2.5. Spatial Distribution

Some metals are considered the most persistent contaminants in groundwater, and once these metals enter an aquifer, the groundwater quality can drastically change [41]. To accurately evaluate the results of the study region, it is necessary to determine spatial and temporal distribution patterns using suitable analytical techniques [42,43]. Numerous research employs appropriate geostatistical approaches for the spatial study of groundwater components [28,44]. In this work, Cr(VI) concentration data were imported into Arc GIS 10.4 and interpolated using the ordinary kriging model in Geostatistical Analyst to forecast the spatial distribution of Cr(VI) concentrations throughout the study area. We set the Transformation type to Log, Order of trend removal to Second, Maximum and Minimum neighbors to 50, and the rest of the parameters to default while using the ordinary kriging model.

## 3. Results

### 3.1. Characterization of Cr(Ⅵ) Contamination

**Figure 2:**
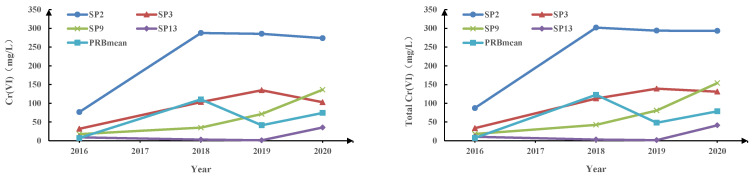
Cr(VI) and total Cr concentrations at typical sampling points and PRB regional means.

The concentrations of Cr(VI) in soil and groundwater samples were obtained in the research region as shown in Table 1 and Table 2, and data is represented in Figure 2. Cr(VI) was detected in all soil samples from the research area, with a maximum value of 566.20 mg/kg and a minimum value of 2.53 mg/kg, while the background level of chromium in typical uncontaminated soil was 0.12 mg/kg, as determined by laboratory analysis [45]. The soil sampling results in the abandoned chemical sites showed that a significant amount of Cr(VI) was present in the soil at the open dump of chromium waste in the study area with a high concentration of 566.2 mg/kg. the remaining five soil sampling sites had Cr(VI) concentrations ranging from 2.53 to 4.17 mg/kg.

The average Cr(VI) concentration in groundwater samples from the research area was 41.99 mg/L, ranging from 0.01 to 464.34 mg/L. The majority of the groundwater samples showed Cr values substantially over the World Health Organization (WHO) drinking water quality threshold, which is 50 μg/L [46]. The study area has a high level of groundwater exploitation, and the survey discovered 39 wells within 2 km^2^ of the Cr open dump site, which are primarily used to irrigate agricultural land. Due to insufficient anti-seepage methods of shallow agricultural irrigation wells in the early years, the short depth of the water table, and intensive groundwater exploitation, shallow water and pressured water have merged, creating a conduit for the downward seepage of Cr (VI). It is observed that the water-soluble Cr from the pollution source primarily contaminates the first restricted aquifer via vertical leaching. Since the Cr in groundwater is driven by the horizontal hydraulic gradient. There is a nearly 20m clay layer between the first confined aquifer and the second aquifer. Therefore, the deep aquifers in the study area are unlikely to be contaminated.

### 3.2. Spatial and Temporal Effects of PRB on Cr(VI)

**Figure 3:**
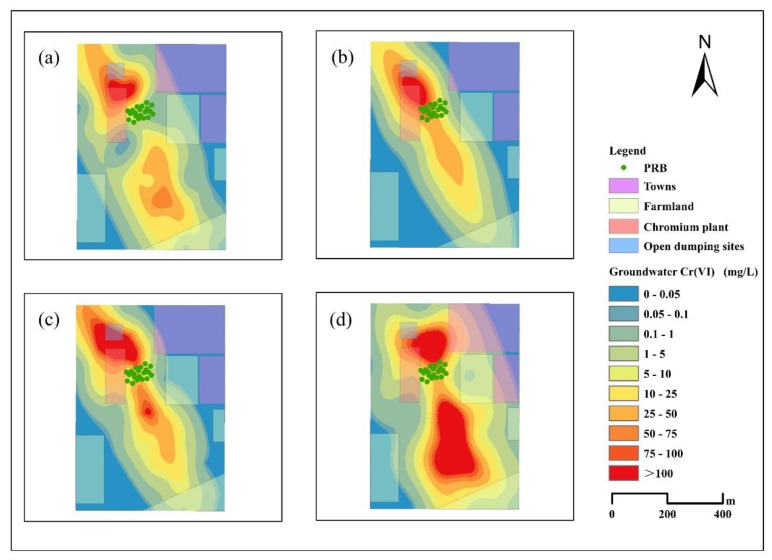
Spatial distribution of Cr(VI) in groundwater (**a**) Concentration distribution in September 2016 (**b**) Distribution of concentrations in April 2018 (**c**) Distribution of concentrations in January 2019 (**d**) Distribution of concentrations in May 2020.

The regional distribution of Cr(VI) in groundwater resources in the research area during four years is depicted in Figure 3 The analysis of the spatial and temporal distribution of Cr(VI) indicates that the highest concentration of Cr(VI) in groundwater is found in the vicinity of the chromium salt production plant, which is consistent with the results of the analysis of Cr(VI) concentration in the soil samples of the study area, which indicates that the soil in the open chromium waste dumps in the study area is the source of Cr(VI) in groundwater. There are only a few sites where the concentration of Cr(VI) in groundwater is below the WHO drinking water limit of 0.05 mg/L and does not threaten human health. Some towns and arable land are already in polluted groundwater zones. During these four years, the extent of Cr(VI) contamination in the research area tended to move southward, possibly due to the direction of groundwater flow and the migration-prone nature of Cr(VI).

In 2016, the study area constructed a PRB to remove Cr(VI) from groundwater. The reaction medium materials in the PRB are ZVI nanoparticles, activated carbon and sand, which have a mass ratio of 3:1:4. Upstream of the PRB, Cr(VI) values ranged from 31.84 to 285.37 mg/L, which is significantly higher than the Cr(VI) levels in contaminated groundwater reported in earlier research [24,47,48]. Figure 2 shows the Cr(VI) concentrations at several representative sampling points within site over the four years and the average Cr(VI) concentrations in the PRB area. Observation in the downstream monitoring well of groundwater flow in the southern part of PRB shows that the Cr(VI) concentration of SP9 is 17.83 mg/L, 35.08 mg/L, 71.43 mg/L and 136.12 mg/L; SP13 is 9.29 mg/L, 3.15 mg/L, 1.30 mg/L and 35.45 mg/L within four years. In comparison, monitoring results on the south side of the PRB show that the concentration of SP2 is 76.52 mg/L, 287.64 mg/L, 285.37 mg/L and 273.88 mg/L; SP3 is 31.8 mg/L, 103.4 mg/L, 134.89 mg/L and 102.91 mg/L. The trend of total Cr concentrations at several typical sampling sites is generally consistent with hexavalent chromium. The overall trend of increasing Cr(VI) and total Cr concentrations in the selected typical sampling points was observed with increasing time. Meanwhile, there is a certain lag in elevated total Cr and Cr(VI) concentrations at typical sampling points downstream of the PRB, which may be due to the slow diffusion of the upstream pollution plume downstream. The results showed that the groundwater north of the PRB contained high concentrations of Cr(VI), which were reduced after remediation by the PRB.

Over the period of four years, the Cr(VI) concentrations in the monitoring wells at the PRB ranged from 0.12 to 27.91 mg/L, 0.05 to 464.34 mg/L, 0.08 to 238.42 mg/L, and 0.04 to 362.42 mg/L, respectively. Meanwhile, Cr(VI) concentrations in the monitoring wells downstream of the PRB varied between 0.04 and 17.83 mg/L, 0.03 and 35.08 mg/L, 0.01 and 71.43 mg/L, and 0.01 and 136.12 mg/L in these four years. Three monitoring wells had Cr(VI) concentrations in excess of 300 mg/L in 2018, all in the PRB area. However, in the subsequent two years of monitoring data, their Cr(VI) concentrations decreased again. The ${\mathrm{PRB}}_{\mathrm{mean}}$ curve in Figure 2 has a significant increase in 2018 and is the maximum of the 4-year average PRB regional concentration. The study area experienced several heavy rainfall events and flooding in 2017. Higher water level fluctuations can raise the maximum concentration of chromium and lead to longer migration distances due to changes in precipitation and meteorological conditions throughout time [49,50]. Our results suggest that heavy rainfall and flooding may have exacerbated Cr(VI) transport in groundwater and contributed to the elevated Cr(VI) concentrations in groundwater in the study area in 2018.

### 3.3. Characterization of the Health Risk

The general screening level for Cr(VI) in industrial sites was 5.7 mg/kg, with 17% of the soil samples exceeding this level, up to 99 times the general screening level. The WHO drinking water standard of 0.05 mg/L was utilized as the screening value for all groundwater samples in the research area, with 88.6% of samples exceeding this threshold. Table 1 displays the carcinogenic risk (CR) and hazards quotient (HQ) statistics for the human health risk assessment of Cr(VI) in soil samples from chemical plants. Table 2 displays the CR and HQ of Cr(VI) in four-years groundwater samples from the study area. Areas with acceptable human contamination have a carcinogenic risk of less than 10^−6^ or a hazard quotient of less than 1 [51]. The open pile included the highest carcinogenic risk of the six soil samples from the chemical plant area, at 9.57 × 10^−6^, while the remaining samples were well below the threshold of 10^−6^. The distribution of non-carcinogenic and carcinogenic risk in soil samples from the research region was consistent, with the open pile’s hazard quotient surpassing 1 by 4.44. Over four-year period, 92.4% of the groundwater samples collected had a carcinogenic risk above 10^−6^, and 85.6% had a hazard quotient larger than 1.

### 3.4. Sensitivity Analysis for Carcinogenic Risk Assessment

Separate Sobol sensitivity assessments were performed for the parameters included in the carcinogenic risk estimations for exposure to soil (ingestion, cutaneous, inhalation) in the vicinity of the chemical plant and drinking groundwater throughout the entire study region. The range of input parameters substantially influences the model’s sensitivity [35]. Table 3 displays the input parameters analyzed during sensitivity analysis, with Sobol sensitivity analysis examining the most influential parameters. Sobol scores are divided into three categories, first-order effects (FOE), second-order effects (SOE), and total effects (TE), respectively [36]. The FOE and TE are used to assess the relative sensitivity of the parameters, while the SOE is used to determine the interaction effects between the various parameters [35].

**Figure 4:**
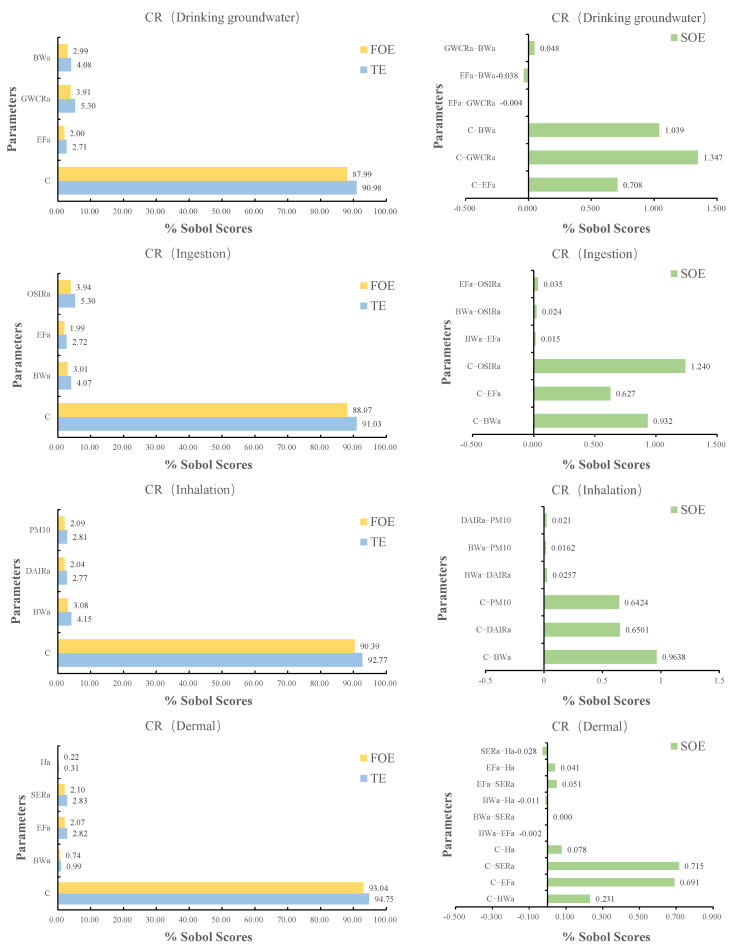
Sensitivity analysis based on CR models for different exposure routes, considering first-order effects (FOE), second-order effects (SOE) and total effects (TE).

As shown in Figure 4 the Sobol scores for the input parameters of the CR model for the various exposure pathways. According to the research, C is the most sensitive metric among the four possible exposure pathways for FOE and TE, with a Sobol score much higher than the other parameters. The second sensitive parameter in the exposure route of drinking groundwater is GWCRa, while the third sensitive parameter is BWa. C-GWCRa > C-Bwa > 1%, showing considerable interaction between GWCRa and BWa. For SOE, C-OSERa > C-BWa > C-EFa, where C-OSERa scores > 1% and all other interaction effect scores are 1%, showing a substantial interaction between OSERa and C. When the carcinogenic risk is posed by pollutants in soil, the sensitivity OSERa is greater than BWa and EFa under the ingestion exposure pathways. Under dermal exposure, SERa was greater than EFa, BWa, and Ha, and C-SERa was greater than C-EFa, but the interaction effect scores were all less than 1%. Under the inhalation route, BWa > PM10 > DAIRa, and for SOE, C-BWa had the greatest interaction score of 0.96%, which did not surpass 1%.

## 4. Discussion

### 4.1. Characterization of Cr(Ⅵ) Contamination

Maximum detection of Cr(VI) in soil samples from the research area occurred near the chromium slag pile, which may explain the high presence of heavy metals in the soil given that the slag was deposited in the open [30]. The periodic leaching of Cr(VI) from chromium slag heaps during seasons of ample water is the cause of soil contamination, which contaminates the study area via direct runoff. In general, samples closest to the source area (chromium salt production plant and chromium slag heap) contained greater amounts of Cr(VI), such as SP2 and SP3. A borehole near the chemical plant at a depth of approximately 40 m detected total Cr and Cr(VI) in the first pressurized aquifer (depth 7.9–11.5 m) and in the clay, layer 0.5 m below the aquifer, with total Cr content reaching up to 297.88 mg/kg (at 11.3 m) and Cr(VI) content reaching up to 79.4 mg/kg (at 12.0 m). This is likely because the reduction of Cr(VI) to Cr(III) happens during the infiltration of Cr(VI) and is promoted by reducing chemicals such as Fe(II) and organic carbon in the soil [49,52], whereas Cr(VI) is more mobile than Cr(III) [53].

### 4.2. Spatial and Temporal Effects of PRB on Cr(VI)

The groundwater in the site was seriously polluted for a long time, and the concentration of Cr(VI) in the groundwater treated by PRB on the south side was significantly reduced during the initial period. Under a natural hydraulic gradient, the target pollutants are degraded, adsorbed, or precipitated as the plume passes through the PRB [54,55]. Studies have demonstrated that a reaction media deposited in the subsurface by a PRB can efficiently convert or immobilize pollutants under specific conditions and over time [21,56]. Metal ions continue to occupy empty spaces on the composite surface as the contact duration between the PRB and the groundwater increases, resulting in a negligible increase in adsorption [57]. The main problems with using ZVI as a reaction medium are the surface precipitation due to the formation of magnetite, ferrous hydroxide, green rust and iron oxyhydroxides, which can decrease the permeability of PRBs and hence cause PRB clogging [58]. During the first few years following the installation of a PRB, clogging is negligible. However, as the clogging progresses toward the outlet, there is a significant change in hydraulic conductivity and porosity, meaning that the PRB will not be able to remove contaminants from the groundwater [59]. The change in Cr(VI) concentrations in the monitoring wells at the PRB is most likely attributable to the operation at high concentrations, which led to the clogging of the PRB unit, a progressive decline in performance, and the ongoing release of Cr(VI) from the soil at the upstream source. In a study of ZVI-PRB groundwater remediation at an abandoned chromium salt plant in China, Cr(VI) concentrations in monitoring wells downstream of the PRB were enhanced 90 days after Cr slags were not removed from the upstream soil package gas zone [24]. Over the period of four years, Cr(VI) concentrations in the monitoring wells downstream of the PRB varied between 0.04 and 17.83 mg/L, 0.03 and 35.08 mg/L, 0.01 and 71.43 mg/L, and 0.01 and 136.12 mg/L. This suggests that as PRB blockage increases and Cr(VI) is released from upstream soils, the effectiveness of PRB in removing Cr(VI) from groundwater decreases and the contamination plume spreads downstream.

Several studies have demonstrated that PRB effectively removes Cr(VI) from contaminated groundwater [24,60]. Under laboratory circumstances, the PRB of some composites removed up to 95% of Cr(VI) after four cycles of use [57]. However, implementation at actual locations has been subpar, with Cr(VI) concentrations exceeding the standard still being monitored downstream in 2016 and the operation of the PRB being substantially hindered by blockage from numerous sources. Chemical and biological blockage can considerably impair the porosity and hydraulic conductivity of the PRB, consequently diminishing its durability [23]. As the performance of the PRB deteriorated due to clogging, the plume began to slowly spread southwards, with the PRB losing nearly all of its ability to prevent Cr(VI) transport in the study area by 2020, with pollution levels downstream broadly consistent with those upstream and significantly exceeding the monitoring results from the previous three years. Irrigation of the agricultural regions via groundwater extraction at the downstream end also contributes to the plume’s conveyance. To prevent further pollution from spreading, the soil and contaminated groundwater of the chrome waste open discharge site should be remedied, and groundwater development in the vicinity should be limited.

### 4.3. Characterization of the Health Risk

The carcinogenic risk and hazard entropy were greatly decreased in groundwater treated with PRB, but significantly elevated when PRB failed. This suggests that Cr(VI) continues to contaminate groundwater in the soil at the open pile in the research region, resulting in unacceptable levels of Cr(VI) in groundwater, which can pose significant carcinogenic and non-carcinogenic threats to human health. The analysis of groundwater samples from the south side of the PRB revealed that despite the effectiveness of the PRB in decreasing carcinogenic and non-carcinogenic risks to humans from the downstream side of the groundwater, the lowered risk was still unacceptable.

The contribution of different routes of exposure to carcinogenic risk in soil showed that inhalation (89.7%) > dermal contact (7.1%) > ingestion (3.2%). Inhalation of airborne soil particles was the primary pathway of carcinogenic risk from Cr(VI) in the soil in the research area, followed by cutaneous exposure, which together accounted for 96.9% of the carcinogenic risk. The study results reveal that both the soil and groundwater in the study region are substantially contaminated, exposing residents to unacceptable levels of carcinogenic health hazards via oral, dermal, inhalation, and drinking groundwater. Therefore, site controls should decrease the risk to human health posed by Cr(VI) by minimizing skin contact with contaminated soil and providing employees with suitable personal protection [61]. In the vicinity of the study area, where groundwater is one of the primary sources of domestic water, long-term drinking and bathing with Cr(VI)-contaminated water can lead to a significant accumulation of chromium in the human body, with potentially severe consequences including apoptosis, DNA damage, and carcinogenesis. In terms of non-carcinogenic risk contribution, cutaneous contact (54.2%) is greater than oral consumption (23.1%) and inhalation (22.7%).

### 4.4. Sensitivity Analysis for Carcinogenic Risk Assessment

The results of the sobol sensitivity analysis reveal that C is the most sensitive parameter across all exposure pathways, with substantial interaction effects with other factors. The subsequent most sensitive measures for drinking groundwater, ingestion, cutaneous exposure, and inhalation are GWCRa, OSERa, SERa, and BWa, respectively. Different regions have reported similar studies. In a study of the effects of fluoride and nitrate in groundwater on human health in India, the groundwater concentration factor was the most influential variable [29]. Another study of Sobol sensitivity analysis of groundwater fluoride intake and cutaneous exposure pathways in southern India revealed that the concentration of fluoride in groundwater was the most sensitive parameter for individuals of all ages and sexes [35]. A sensitivity analysis of heavy metals in groundwater for health risk assessment revealed, in a study on hazardous heavy metals in groundwater in southern Iran, that the most useful parameter for children and adults was the concentration of the contaminant [32].

## 5. Conclusions

The results of the study indicate that the soil at the chromium slag heap is a source of Cr(VI) in groundwater and continues to contaminate groundwater in the study area. The PRB effectively removed Cr(VI) from groundwater during the initial period, but the removal was limited. As PRB blockage increases and Cr(VI) is released from upstream soils, the effectiveness of PRB in removing Cr(VI) from groundwater decreases and the contamination plume spreads downstream. By 2020, the level of contamination downstream of the PRB will be almost the same as that upstream. In general, the carcinogenic and non-carcinogenic hazards for the six soil samples at the open dump site were 8.77 × 10^−5^ and 4.44, respectively, and the rest fell within the acceptable range. The contribution of distinct soil exposure routes to carcinogenic risk was as follows: inhalation (89.7%) > dermal contact (7.1%) > oral ingestion (3.2%). For non-carcinogenic risks, dermal contact (54.2%) > oral intake (23.1%) > inhalation (22.7%). In groundwater, 92.4% of samples had a carcinogenic risk (CR > 10^−6^), and 85.6% had a hazard quotient (HQ > 1). The findings of the sensitivity analysis demonstrated that the concentration of the contaminant (C) was the most sensitive parameter among all exposure paths and that it had substantial interaction effects with other parameters.

The study concludes that Cr(VI) contamination from the open dumps of chromium waste in the former chemical plant in the study area is still severe and poses a threat to human health in the plant area, while residual Cr(VI) in the soil continues to contaminate groundwater in the study area, resulting in Cr(VI) concentrations in groundwater in the study area exceeding the standard and rendering it unfit for human consumption. A properly working PRB at the plant site may efficiently remove Cr(VI) from contaminated groundwater, decreasing both the carcinogenic and non-carcinogenic risks to humans provided by groundwater on the downstream side, but the reduced risk is still unacceptable. Possible blockage in a persistently highly contaminated environment results in poor PRB remediation. Therefore, it is recommended that the contaminated soil and groundwater in the research area be remedied, and the decision authority should take action to prevent the migration of Cr(VI) in groundwater from continuing to pose health risks to adjacent inhabitants.

## Figures and Tables

**Table 1:** Total Cr and Cr(VI) concentrations, carcinogenic and non-carcinogenic risks in soil samples from chemical plants in the study area in 2019.

	Concentration (mg/kg)		CR				HQ			
	Total Cr	Cr (VI)	Ingestion	Dermal Contact	Inhalation	Total CR	Ingestion	Dermal contact	Inhalation	Total HQ
Max	592.81	566.2	2.73 × 10^−6^	6.76 × 10^−6^	7.82 × 10^−5^	8.77 × 10^−5^	1.0068	2.4978	0.9378	4.4423
Min	2.58	2.53	1.22 × 10^−8^	3.02 × 10^−8^	3.49 × 10^−7^	3.92 × 10^−7^	0.0045	0.0112	0.0042	0.0199
Mean	102.04	97.3	4.69 × 10^−7^	1.16 × 10^−6^	1.34 × 10^−5^	1.51 × 10^−5^	0.173	0.4292	0.1611	0.7634

**Table 2:** Total Cr and Cr(VI) concentrations, carcinogenic and non-carcinogenic risks in groundwater samples from the study area at different periods.

Periods	Total Cr (mg/L)			Cr (VI) (mg/L)			CR			HQ		
	Max	Min	Mean	Max	Min	Mean	Max	Min	Mean	Max	Min	Mean
2016.09	87.23	0.01	9.07	76.52	0.01	7.85	3.68 × 10^−3^	4.82 × 10^−7^	3.78 × 10^−4^	1360.3	0.18	139.54
2018.04	487.56	0.04	82.29	464.34	0.03	70.48	2.23 × 10^−2^	1.4410^−6^	3.39 × 10^−3^	8250.69	0.53	1253.22
2019.01	293.86	0.01	40.72	285.15	0.01	35.61	1.37 × 10^−2^	4.82 × 10^−7^	1.72 × 10^−3^	5073.11	0.18	633.28
2020.05	476.51	0.01	64.56	362.42	0.01	54.03	1.75 × 10^−2^	4.82 × 10^−7^	2.60 × 10^−3^	6444.36	0.18	960.72

**Table 3:** Parameters considered for the risk evaluation using Sobol sensitivity approach in the present study. The parameters were selected based on the Technical Guidelines for Risk Assessment of Soil Contamination of Land for Construction and statistical bulletins published by the local authorities and the SD is ±15% of these values.

Parameters	Unit	Value ± SD
Body weight of adults(BWa)	kg	64.2 ± 9.63
Daily groundwater consumption rate of adults(GWCRa)	L·d^−1^	1 ± 0.15
Exposure frequency of adults(EFa)	d·a^−1^	250 ± 37.5
Skin exposure ratio of adults(SERa)	-	0.18 ± 0.027
Concentration(C)	mg/L	min:0.01 max:464.3
Daily oral ingestion rate of soils of adults(OSIRa)	mg·d^−1^	100 ± 15
Height of adults(Ha)	cm	min: 161.5 max: 185
Content of inhalable particulates in ambient air(PM10)	mg·m^−3^	0.119 ± 0.018
Daily air inhalation rate of adults(DAIRa)	m^3^·d^−1^	14.5 ± 2.175

## Data Availability

All the data used for the study appear in the article.
